# Application of double layered end-to-end anastomosis with continuous manual suture for completing digestive tract reconstruction in totally laparoscopic distal gastrectomy

**DOI:** 10.1186/s12893-021-01207-1

**Published:** 2021-04-26

**Authors:** XinSheng Zhang, WeiBin Zhang, MengLang Yuan, XiaoMeng Shi, HongYi Chen, Zhen Feng, ZiHao Chen, DunBo Liu, EnJun Yan, ShuangYi Ren

**Affiliations:** 1 Department of General Surgery, The Second Affiliated Hospital of Dalian Medical University, Zhongshan Road 467, Shahekou District, Dalian, Liaoning China; 2 General Surgery Department of Jinzhou Central Hospital, No.51, Section 2, Shanghai Road, Guta District, Jinzhou, Liaoning China

**Keywords:** Distal gastric cancer, Continuous manual suture, Digestive tract reconstruction, Totally laparoscopic distal gastrectomy, Double layered end-to-end anastomosis with continuous manual suture

## Abstract

**Background:**

We retrospectively reviewed and consecutively collected the clinical data of distal gastric cancer patients who received surgical treatment, and we discuss the safety and feasibility of double layered end-to-end anastomosis with continuous manual suture to complete digestive tract reconstruction in totally laparoscopic distal gastrectomy.

**Methods:**

We reviewed the clinical data of 41 patients with distal gastric cancer from the gastroenterology department of the Second Affiliated Hospital of Dalian Medical University, from September 2018 to August 2019, who underwent totally laparoscopic distal gastrectomy. During the operation, the method of double layered end-to-end anastomosis with continuous manual suture was used for Billroth type I anastomosis to complete digestive tract reconstruction. All patients have been given a follow-up visit and gastroscopy three months after the operation. The peri-operative clinical information and postoperative follow-up information were collected for analysis, and the clinical application value was evaluated.

**Results:**

General information: male(n = 27), female(n = 14), age = 65.02(SD 9.94) years, and BMI = 23.52(SD 2.56) kg/m^2^, Tumor location: antrum(32,78.0%), angle (6,14.6%), and body (3,7.3%). Clinical stage: I (27, 65.9%), II (7, 17.1%), and III (7, 17.1%). Operative information: operation time = 154.51(SD 33.37) min, anastomosis time = 26.88(SD 5.11) min; intraoperative bleeding = 66.34(SD 48.81) ml; first postoperative ambulation Median = 1(IQR 0) d, first postoperative flatus Median = 3(IQR 2) d, first postoperative diet Median = 3(IQR 1) d, postoperative hospital stay Median = 7(IQR 2) d, and total hospitalization cost = 10,935.00(SD 2205.72)USD. Differentiation degree: high and high-moderate (3,7.32%), moderate and poor-moderate (24, 58.54%), poor differentiation (14, 34.15%), dissected lymph nodes Median = 31(IQR 17), and positive lymph nodes Median = 0(IQR 1). Pathological stage: IA (20, 48.78%), IB (3, 7.32%), IIA (4, 9.76%), IIB (5, 12.20%), IIIA (1, 2.44%), IIIB (3, 7.32%), and IIIC (5, 12.20%). Complications (n = 4): lung infection (1, 2.44%), anastomotic leakage (1, 2.44%), and gastroparesis (2, 4.88%).

**Conclusion:**

It is safe and feasible in clinical treatment to apply the method of double layered end-to-end anastomosis with continuous manual suture to complete digestive tract reconstruction in totally laparoscopic distal gastrectomy.

## Background

In recent years, with the advanced development of laparoscopic techniques, totally laparoscopic gastrectomy has been suggested to be safe and feasible, especially, totally laparoscopic distal gastrectomy (TLDG) [1]. The choice of the ideal method for digestive tract reconstruction in distal gastrectomy is still controverial. Various methods of anastomosis with a stapler have been heavily reported in previous studies, such as using linear staplers to perform delta-shaped anastomosis, Billroth II anastomosis or Roux-en-Y anastomosis [2–4]. However, there are still many problems, such as high anastomotic tension, inaccurate tumour margins and large expenses. Therefore, we started to perform totally laparoscopic hand-sewn Billroth I anastomosis with a new application of double layered end-to-end anastomosis with continuous manual suture to complete digestive tract reconstruction, and we have completed 41 cases thus far. We intend to analyse these cases and discuss the relevant issues about of this the new application in totally laparoscopic distal gastrectomy based on previously published studies and our own experiences.

## Methods

### Materials

We retrospectively reviewed and consecutively collected the clinical data of 41 patients with distal gastric cancer from the gastroenterology department of the Second Affiliated Hospital of Dalian Medical University, from September 2018 to August 2019, who underwent totally laparoscopic distal gastrectomy. During the operation, the method of double layered end-to-end anastomosis with continuous manual suture was used for Billroth I type anastomosis to complete digestive tract reconstruction.

#### 1.1 Inclusion criteria

(1) Electronic gastroscopy and biopsy pathological diagnoses were used to confirm distal gastric cancer before surgery; (2) Chest and abdominal computed tomography (CT) scans confirmed no distant metastasis before surgery; and (3) No serious complications of the heart, lungs or other important organs.

#### 1.2 Exclusion criteria

(1) Severe cardiopulmonary cerebral dysfunction, resistant to general anaesthesia and surgery: refractory hypertension, cardiopulmonary dysfunction, respiratory failure, chronic obstructive pneumonia etc.; and (2) Severe liver, renal and coagulation dysfunction; (3) Other operation methods.The operation was performed by the same senior doctors. The patients and their families signed informed consent forms related to the operation. This study was approved by the Institutional Review Board of The Second Affiliated Hospital of Dalian Medical University.

### Methods

**Fig. 1:**
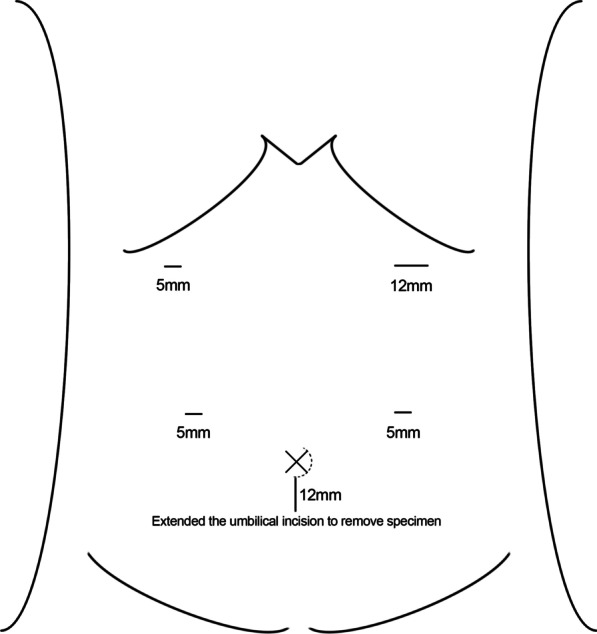
Placement of trocars. The figure depicted in Fig. 1 was drawn by our own. Two 12 mm and three 5 mm trocars were used in operation. The 12 mm trocar below the umbilicus is used as the observation hole, and another 12 mm trocar is the main operation hole

2.1. No gastric tubes were placed before any of the operations. All the operations were performed using the German Braun AESCULAP 3D HD laparoscopic system. The anesthesia method is intravenous-inhalation combined anesthesia. The operation position is head high and foot low (tilt 15°). And trocar position of the puncture are shown in Fig. 1. According to the 15th edition of the Japanese Classification of Gastric Carcinoma, laparoscopic distal gastrectomy and D2 lymph node dissection were performed.
2.2. Digestive tract reconstruction.

**Fig. 2:**
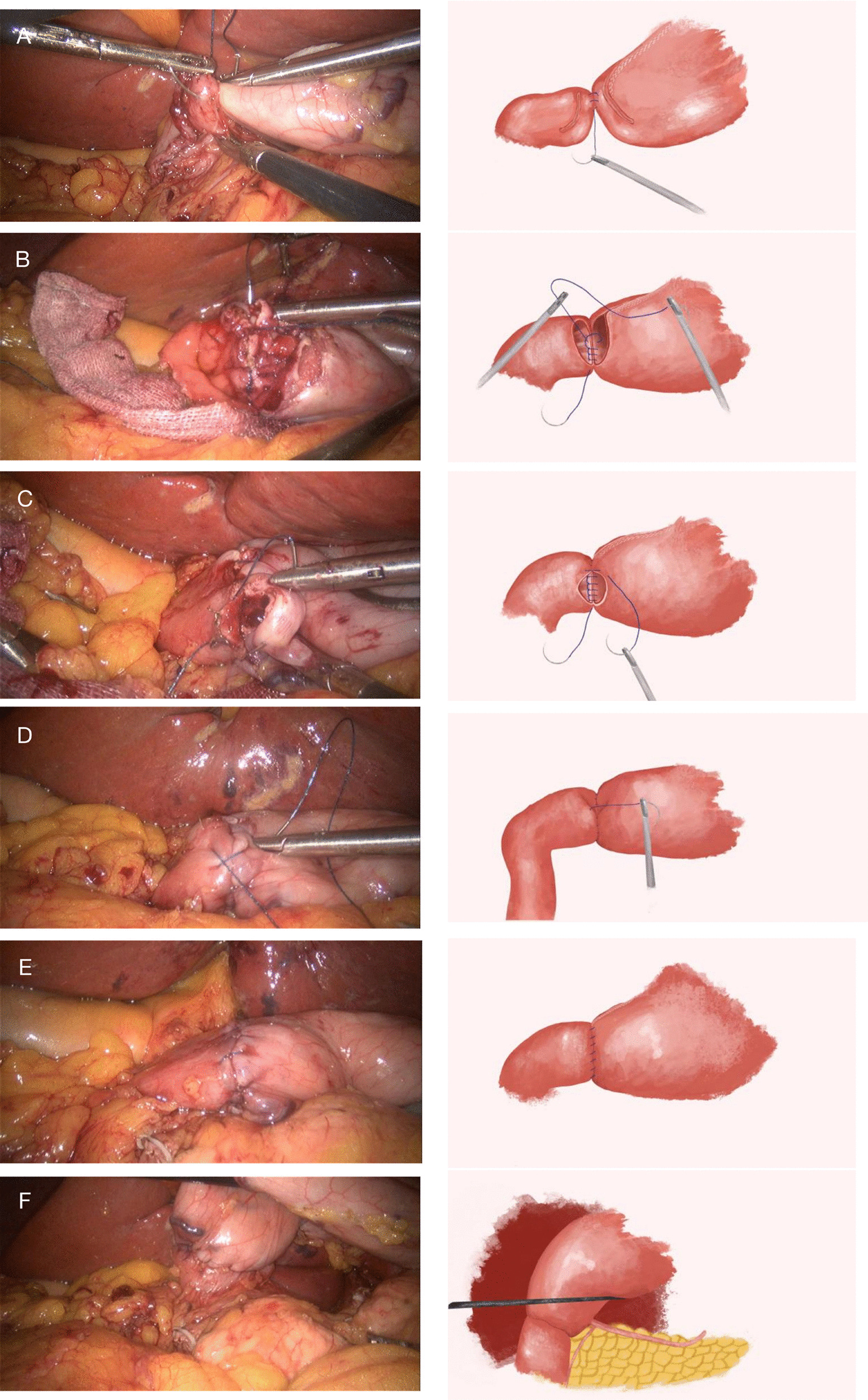
Steps of double layered end-to-end anastomosis with continuous manual suture to complete digestive tract reconstruction in TLDG (Video capture of anastomotic procedure and sketch of anastomosis). **a** The first absorbable barbed thread with single needle(Covidien VLOCL0614, 3-0) to continuous suture of sarcoplasmic layer of the posterior wall from the top to the bottom, and the needle was kept for standby. **b** The second absorbable barbed thread with single needle (Covidien VLOCL0614, 3-0) to continuous lock-stitch suture, from bottom to top, of the whole layer from the posterior wall to anterior wall. **c** The second needle to Connell suture of the whole layer of the anterior wall of the anastomosis. **d** The first standby needle to continuous suture of sarcoplasmic layer of the anterior wall. **e** Display of anastomotic anterior wall. **f** Display of anastomotic posterior wall.

2.2.1. Anastomotic placement.
  The duodenum and the greater curvature of the remnant stomach were occluded with interdiction clamps located approximately 2 cm away from the predetermined position of the anastomotic opening to block the reflux of digestive fluid.
2.2.2 The first single needle for continuous suture of the sarcoplasmic layer of the posterior wall.
  First, if necessary, the upper and lower margins of the posterior duodenal wall were sewn with one stitch each to the seromuscular layer of the posterior gastric wall to obtain relative immobilization between the duodenal stump and the remnant stomach, Then, the posterior wall of the remnant stomach and the posterior wall of the duodenum were sutured continuously from top to bottom with an absorbable barbed thread with single needle (Covidien VLOCL0614, 3-0), and the needle was kept for standby.
2.2.3. The second single needle for continuous lock-stitch suture of the whole layer from the posterior wall to the anterior wall.
  The whole layer of the duodenal stump and the remnant stomach were cut through with ultrasonic dissector, and those sizes were equal to the width of the duodenum, which were as the two ends of the anastomosis. The second absorbable barbed thread with single needle (Covidien VLOCL0614, 3-0) was used for continuous lock-stitch suture, from bottom to top, of the whole layer of the posterior wall of the anastomosis. Then, the Connell suture method was adopted to sew the whole layer of the anterior wall of the anastomosis until the starting point of the second single needle, and the anastomosis was completely closed.
2.2.4 The first standby needle for continuous suturing of the sarcoplasmic layer of the anterior wall.
  From bottom to top, the sarcoplasmic layer anastomosis of the anterior wall was continuously sutured with the first standby needle to completely embed the sarcoplasmic layer of the anastomosis.
2.2.5 Release of the interdiction clamps.
  The interdiction clamps were released on both sides of the anastomosis, and the reconstruction of the digestive tract was completed. If necessary, an electronic gastroscope was used to check the internal patency of the anastomosis and whether there were bleeding or weak points.
  See Fig. 2 for details.

### Observation index

3.1. General information: age, sex, BMI, previous abdominal surgery, tumor location, and clinical stage.
3.2. Surgical information: operation time, anastomosis time, intraoperative bleeding; tumour diameter, tumor margin, dissected lymph node, first postoperative ambulation, first postoperative flatus, first postoperative diet, postoperative hospital stay, total hospitalization cost, differentiation degree, pathological stage, and complications.
3.3. Postoperative follow-up information: we performed an upper gastrointestinal imaging at 6 days, an upper gastrointestinal imaging and a gastroscopy at 1 month, and a gastroscopy at 3 months after the operation in all of the patients.

### Fitting equation and CUSUM analysis

All patients were arranged according to the operation sequence and calculated according to the equation: $\mathrm{CUSUM}=\sum_{\mathrm{i}=1}^{\mathrm{n}}(\mathrm{Xi}-\upmu )$, Xi represents the operation time or anastomosis time of each patient, $\upmu$ represents the corresponding mean time, and n represents the serial number of the patient.

Draw scatter diagram of learning curve: the operation sequence as horizontal axis and CUSUM value as vertical axis. SPSS 24.0 statistical software was used to fit the learning curve. The fitting model test was based on *P* value, *P* < 0.05 as the standard of curve fitting success. The goodness of fit is determined by coefficient *R*^2^, that is, the closer *R*^2^ is to 1, the higher the goodness of fit is, and the model with the largest R^2^ is selected as the best fitting model. The falling point in the curve represents the starting point of the case data lower than the mean value, and the corresponding horizontal axis is the number of surgical cases needed to cross the learning curve.

### Statistical method

All data analysis was performed using SPSS 24.0 statistical software. The count data were measured by χ^2^ tests, the normal distribution measurement data were described as‾x ± s, and the skewed distribution measurement data were described as the Median (IQR). The standard level α was 0.05,and the difference was statistically significant when *p* < 0.05.

## Results

### General information

**Fig. 3:**
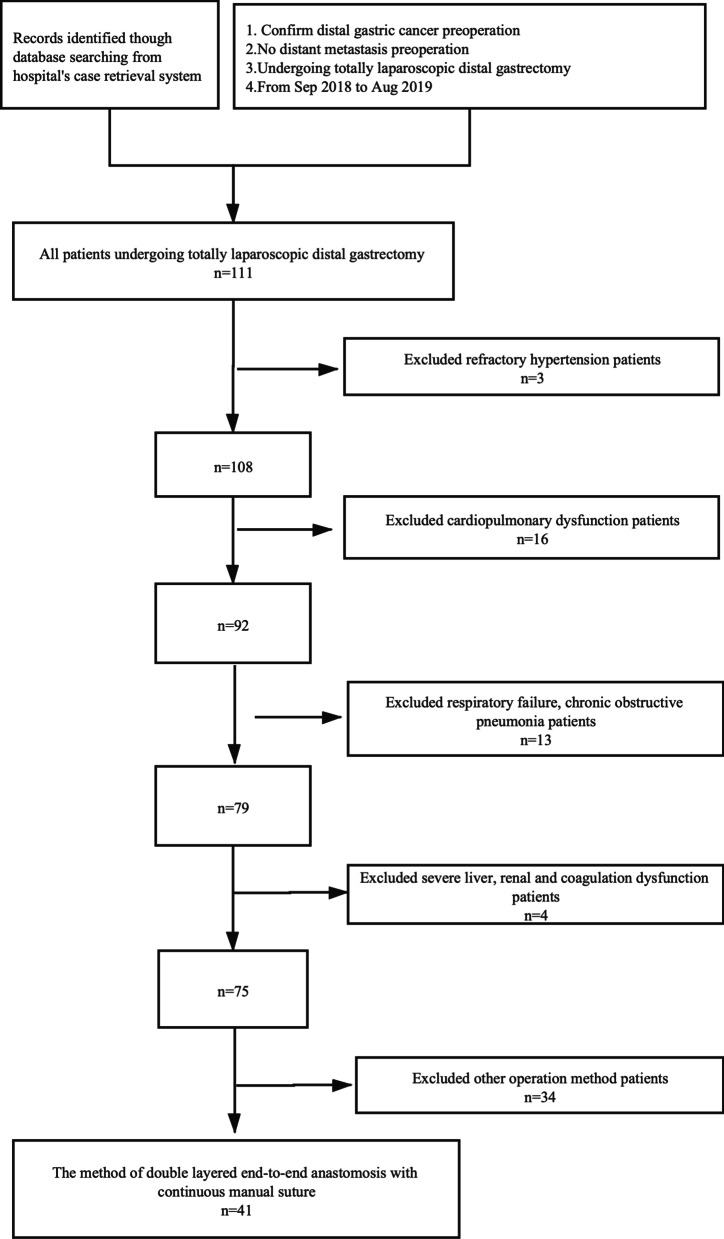
A flow diagram for the study selection. Our records identified though database searching from hospital's case retrieval system, according to the search conditions: 1. Confirm distal gastric cancer preoperation; 2.No distant metastasis preoperation; 3.Undergoing totally laparoscopic distal gastrectomy; 4.From Sep 2018 to Aug 2019. The total number of patients undergoing totally laparoscopic distal gastrectomy in the study period was 111. 70 cases of those that do not meet the requirements were excluded one by one, according to the exclusion criteria: refractory hypertension, cardiopulmonary dysfunction, respiratory failure, chronic obstructive pneumonia etc., severe liver, renal and coagulation dysfunction, and other operation method. Finally, 41 cases were included

Our records identified though database searching from hospital's case retrieval system, according to those search conditions: 1. confirm distal gastric cancer preoperation; 2.no distant metastasis preoperation; 3.undergoing totally laparoscopic distal gastrectomy; 4.from Sep 2018 to Aug 2019. The total number of patients undergoing totally laparoscopic distal gastrectomy in the study period was 111. 70 cases of those that did not meet the requirements were excluded one by one, according to the exclusion criteria: refractory hypertension, cardiopulmonary dysfunction, respiratory failure, chronic obstructive pneumonia, renal insufficiency, other operation method. Finally, 41 cases were included. A flow diagram has been added(Fig. 3).

A total of 41 patients were included in this study: 27 males and 14 females. The mean age was (65.02 ± 9.94) years. The mean BMI was (23.52 ± 2.56) kg/m^2^.The tumour location was distributed as follows: antrum (32, 78.0%), angle (6, 14.6%), and body (3, 7.3%). The tumour clinical stage was distributed as follows: I (27, 65.9%), II (7, 17.1%), III (7, 17.1%).

As shown in Table 1.

**Table 1:** General Information

Index	n = 41
Age(years)	65.02 ± 9.94
Sex(M/F)	27/14
BMI(kg/m^2^)	23.52 ± 2.56
Previous abdominal surgery(n)	2 (13.3)
Tumor location	
Antrum	32 (78.0)
Angle	6 (14.6)
Body	3 (7.3)
Clinical stage	
I	27 (65.9)
II	7 (17.1)
III	7 (17.1)
IV	0

The data were expressed as mean ± SD or number (%)

*F* female, *M* male

### Surgical Information

The operations were smooth, and no serious complications occurred in the perioperative period.

**Fig. 4:**
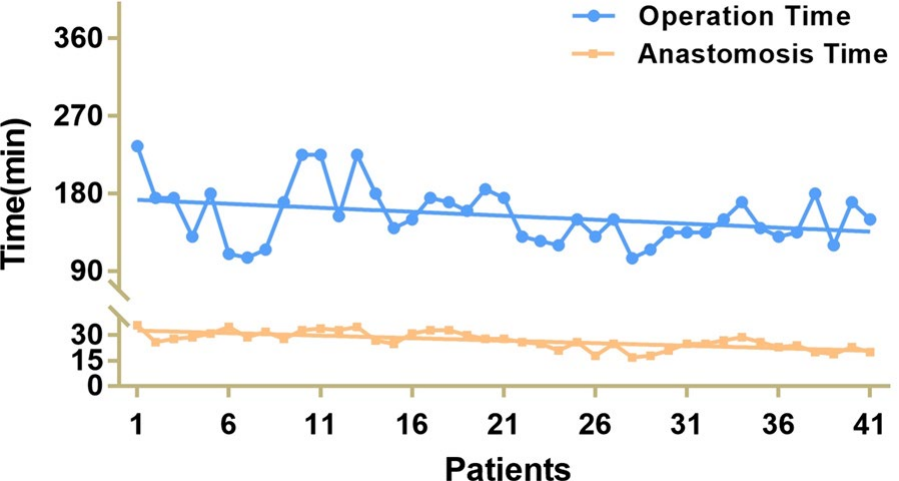
Time of the total operation and the hand-sewn anastomosis of each patient. The hand-sewn anastomosis procedural time was defined as the time from suturing the seromuscular layer of posterior duodenal wall and gastric wall to the seromuscular suture completed of the anterior wall of anastomotic stoma

The mean duration of the operation was (154.51 ± 33.37) min and the mean time to complete the anastomosis was (26.88 ± 5.11) min, as shown in Fig. 4. The amount of intraoperative bleeding was (66.34 ± 48.81) ml, and the mean tumor diameter was (3.07 ± 1.68) cm. The proximal tumor margin was 5.09 ± 1.47 cm. The distal tumour margin was (3.23 ± 1.28) cm. The number of dissected lymph nodes was Median = 31(17), and the number of positive lymph nodes was Median = 0 (1). The differentiation degree was distributed as follows: high and high-moderate (3,7.32%), moderate and poor-moderate (24, 58.54%), and poor differentiation (14, 34.15%). In terms of the postoperative pathological TNM stage, the results showed that there were 23 patients in stage I: IA (20, 48.78%), and IB (3, 7.32%); 9 patients in stage II: IIA (4, 9.76%) and IIB (5, 12.20%); and 9 patients in stage III: IIIA (1, 2.44%), IIIB (3, 7.32%), IIIC (5, 12.20%).

Of the 41 patients, no serious complications occurred, but four controllable complications occurred: one case of postoperative pulmonary infection (1, 2.44%), one case of anastomotic leakage (1, 2.44%), and two cases of gastroparesis (2, 4.88%).According to Clavien Dindo classification, postoperative complications were evaluated, including Grade I(3, 7.3%), Grade II(1, 2.4%). All the above patients recovered after conservative nonoperative treatment.

All patients were able to get out of bed the first day after the operation. The first postoperative ambulation occurred at Median = 1(0) days, and the first postoperative flatus occurred at Median = 3(2) days. The first postoperative diet occurred at Median = 3(1) days. The postoperative hospital stay was Median = 7(2) days, and the total hospitalization cost was 10,935.00 ± 2205.72USD.

As shown in Table 2.

**Table 2:** Intraoperative and postoperative condition [means ± standard deviations, Median (IQR), n (%)]

Index	n = 41
Operation time (min)	154.51 ± 33.37
Anastomosis time^a^ (min)	26.88 ± 5.11
Intraoperative bleeding (ml)	66.34 ± 48.81
Maximal tumor diameter (cm)	3.07 ± 1.68
Proximal tumor margin (cm)	5.09 ± 1.47
Distal tumor margin (cm)	3.23 ± 1.28
Dissected lymph node (Median)	31 (17)
Positive lymph node (Median)	0 (3)
First postoperative ambulation (Median)	1 (0)
First postoperative flatus (Median)	3 (2)
First postoperative diet (Median)	3 (1)
Postoperative hospital stay (Median)	7 (2)
Total hospitalization cost (USD)	10,935.00 ± 2205.72
Differentiation degree	
High and high-moderate differentiation	3 (7.32)
Moderate and poor-moderate differentiation	24 (58.54)
Poor differentiation	14 (34.15)
Pathological stage	
IA	20 (48.78)
IB	3 (7.32)
IIA	4 (9.76)
IIB	5 (12.20)
IIIA	1 (2.44)
IIIB	3 (7.32)
IIIC	5 (12.20)
IV	0
Complications	
Lung infection	1 (2.44)
Anastomotic leakage	1 (2.44)
Gastroparesis	2 (4.88)

The data were expressed as mean ± SD, Median(IQR)or number (%), as appropriate

^a^The time from suturing the seromuscular layer of posterior duodenal wall and gastric wall to the seromuscular suture completed of the anterior wall of anastomotic stoma

### Postoperative follow-up information

**Fig. 5:**
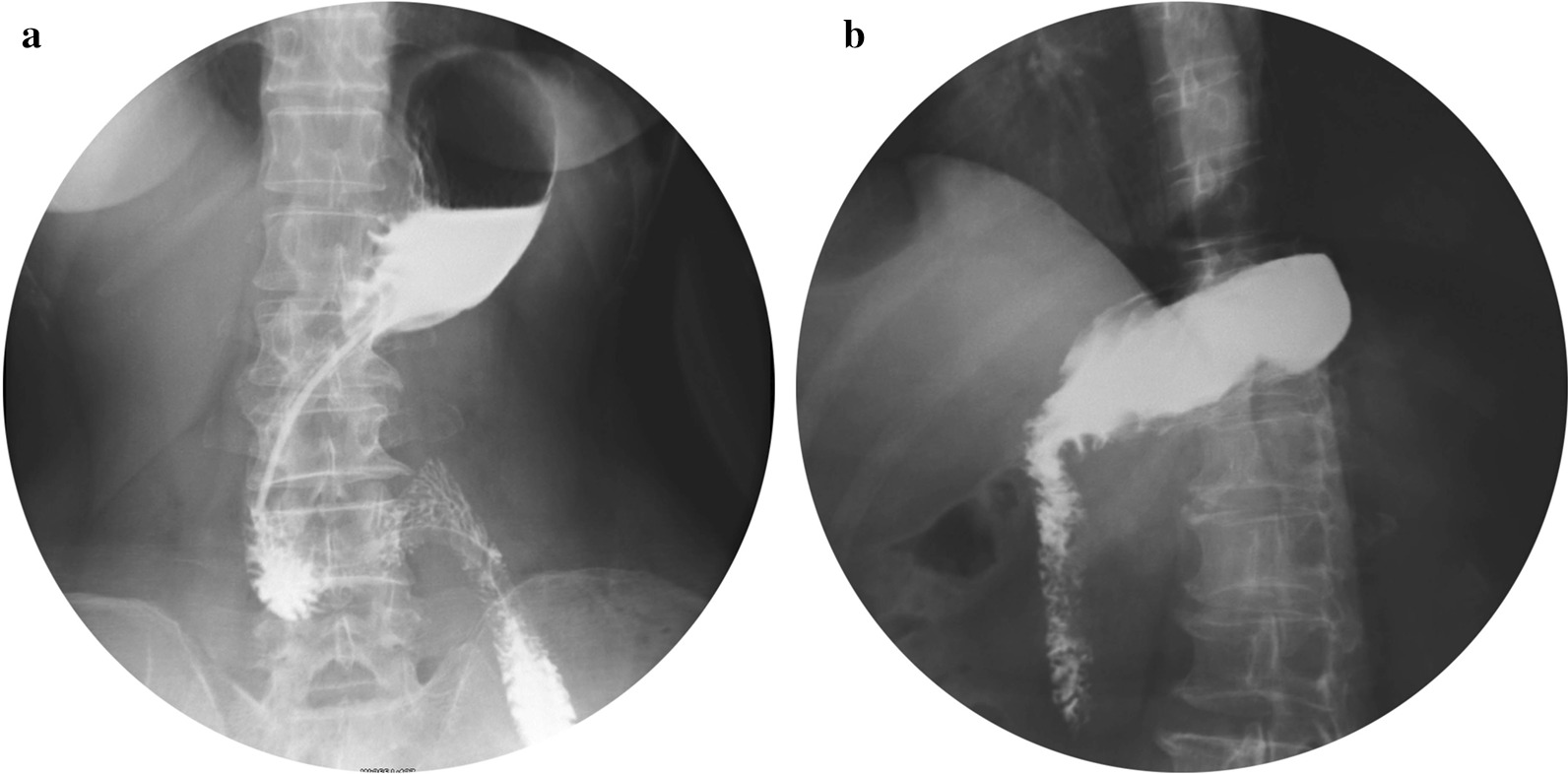
Upper gastrointestinal imaging 6 days and 1 month after operation. **a** X-ray performed 6 days after the operation shows that the anastomotic stoma was unobstructed and there was no anastomotic leakage. **b** X-ray imaging performed 1 month after the operation

**Fig. 6:**
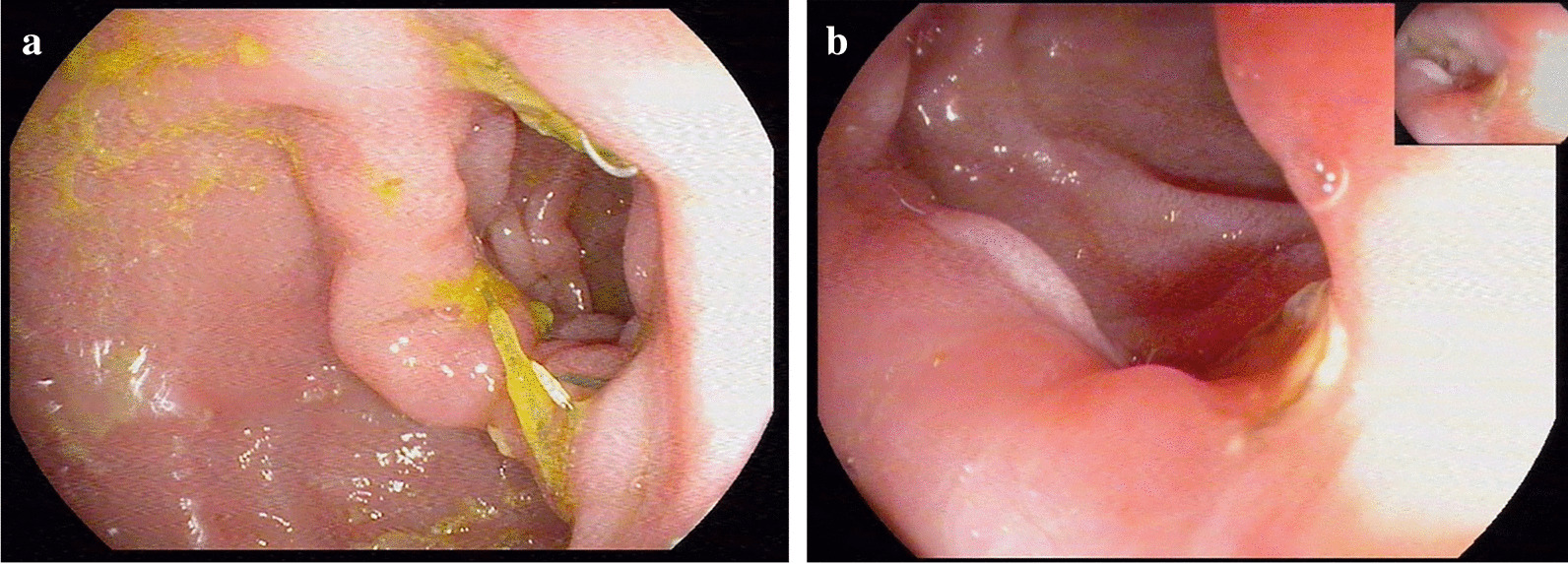
Gastroscopic images of anastomosis 1 months and 3 months after the operation. **a** Gastroscopic view at postoperative 1 months after the hand-sewn anastomosis procedure. **b** Gastroscopic view at postoperative 3 months after the hand-sewn anastomosis procedure

All the patients returned for visits in the outpatient department during the 1st and 3rd months after the operation. All patients exhibited a satisfactory appetite, and there were no symptoms of discomfort such as abdominal pain or bloating. Upper gastrointestinal imaging showed that the anastomosis was unobstructed, as the contrast agent smoothly entered the duodenum (Fig. 5). Gastroscope examination 3 months after the operation showed that the anastomosis had healed well and that there was no stenosis (Fig. 6).

### CUSUM analysis

**Fig. 7:**
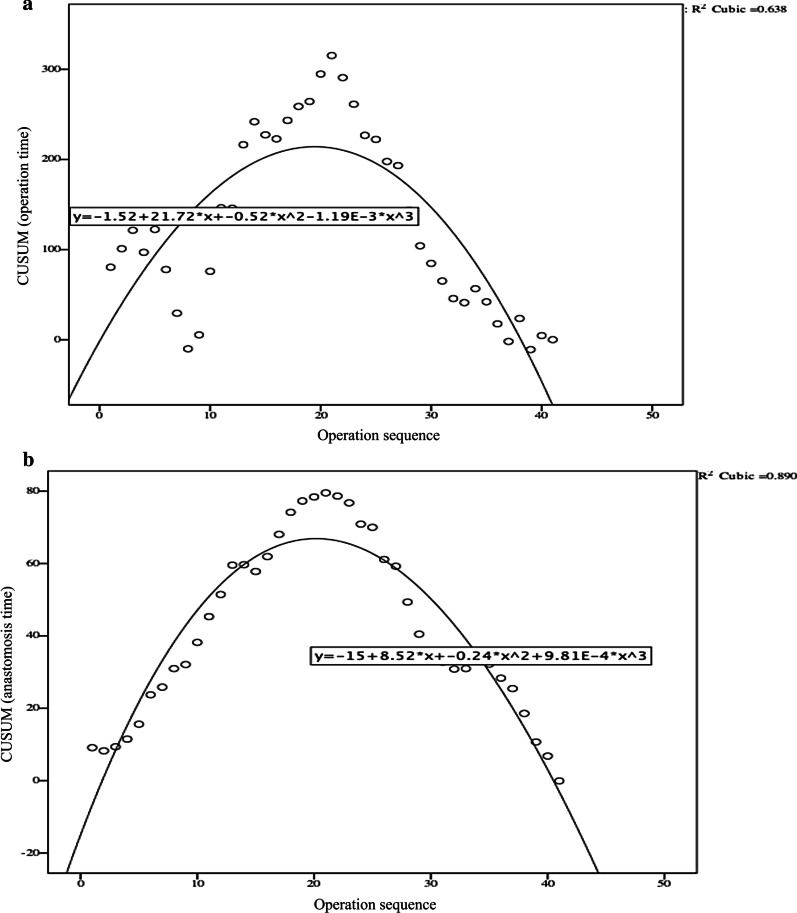
CUSUM analysis for operation time and anastomosis time. The operation sequence as horizontal axis and CUSUM value as vertical axis. The fitting model test was based on *P *value, *P* < 0.05 as the standard of curve fitting success. The goodness of fit is determined by coefficient *R*^2^, that is, the closer *R*^2^ is to 1. **a** CUSUM analysis for operation time. **b** CUSUM analysis for anastomosis time

The best fitting equation of CUSUM learning curve was: CUSUM (operation time) = − 1.52 + 21.72*X−0.52*X^2^−1.19*10^–3^*X^3^, CUSUM( anastomosis time) = −15 + 8.52*X−0.24*X^2^ + 9.81*10^–4^*X^3^ ( X means surgical cases), the P value of fitting model test were < 0.05, with goodness of fit (R^2^) as 1. CUSUM (operation time) reached the peak when the number of surgical cases accumulated to the 21th case, and 21 cases were the minimum number of surgeries needed to cross the learning curve. As the same way, CUSUM (anastomosis time) reached the peak when the number of surgical cases accumulated to the 21th case, and 21cases were the minimum number of surgeries needed to skillfully master double layered end-to-end anastomosis with continuous manual suture to complete digestive tract reconstruction across the learning curve(Fig. 7).

## Discussion

Totally laparoscopic gastrectomy has shown distinct advantages compared to laparoscopic-assisted gastrectomy [4–6]. In totally laparoscopic distal gastrectomy, digestive tract reconstruction has been a key and difficult part of surgery. At present, various anastomotic methods have their own advantages and disadvantages. For patients with lower gastric cancer, most of them undergo delta-shaped anastomosis, Billroth II anastomosis or Roux-en-Y anastomosis with a stapler [1, 7, 8]. Billroth I anastomosis is considered to be more in line with human physiology and anatomy, so it has been favour by surgeons. To date, triangle-shaped anastomosis or modified triangle-shaped anastomosis with a stapler is the most commonly chosen kind of Billroth I anastomosis [7, 9]. Totally laparoscopic triangle-shaped anastomosis with a stapler requires overlapping the remnant stomach wall and duodenum, theoretically wasting part of the remnant stomach wall and duodenal wall and increasing the anastomotic tension. At the same time, the dissection of tumours located higher or near the gastric body is prone to causing insufficient margins, thus greatly limiting the indications for totally laparoscopic Billroth I anastomosis [10]. Billroth II anastomosis can lead to reflux gastritis due to bile reflux, which can increase the incidence of remnant gastric cancer [11]. As a result, this procedure has fallen out of favour for digestive tract reconstruction. Roux-en-Y anastomosis requires two anastomoses, thus increasing the number of intestinal stumps, and requires rather complicated steps. Additionally, similar to Billroth II anastomosis, Roux-en-Y anastomosis does not highly conform to human physiology and anatomy [8, 12]. If postoperative complications of biliary tract diseases such as bile duct stones occur, ERCP and other tests cannot be performed. Totally laparoscopic hand-sewn Billroth I anastomosis can avoid the shortcomings of both Roux-en-Y anastomosis and Billroth II anastomosis.

### Indications

At present, there remain no published studies at home or abroad on hand-sewn Billroth I anastomosis for digestive tract reconstruction after totally laparoscopic distal gastrectomy for the treatment of lower gastric cancer. Our team developed a hand suture technique to directly perform standard end-to-end anastomosis of the remnant stomach and duodenal stump. Similar to hand-sewn Billroth I anastomosis in open gastrectomy, this method could reserve partial walls of the remnant stomach and duodenum compared with triangle-shaped anastomosis with a stapler, ensuring sufficient tumour margins and radical dissection of tumour tissues. Therefore, operative indications for totally laparoscopic Billroth I anastomosis can be greatly broadened, and the difficulty of the surgery can ultimately be reduced. Our team concluded that indications for totally laparoscopic hand-sewn Billroth I anastomosis should be equivalent to those for traditional Billroth I anastomosis in open gastrectomy under the mature cooperation of skilled surgeons.

### Safety and feasibility

In our study, the mean duration of the operation was 154.51 ± 33.37 min and the mean time to complete the anastomosis was 26.88 ± 5.11 min (Fig. 4). The amount of intraoperative bleeding was 66.34 ± 48.81 mL. These data indicate that manual reconstruction of digestive tract is feasible [13]. Lymph node dissection was performed strictly according to standard radical (D2) lymphadenectomy. The pathological examination of the proximal and distal margins of the specimens was negative, and the tumour-free distance of both proximal and distal margins was within the required range (Table 2). Therefore, radical dissection could be guaranteed. Gastrointestinal imaging showed normal gastric emptying on the 6th postoperative day (Fig. 5). Only 1 patient developed pulmonary infection and received anti-infective treatment. One patient developed anastomotic leakage, received unobstructed surgical drainage, received placement of a nasoenteral nutrition tube for enteral nutrition support, and recovered 2 weeks later. There were two patients with gastroparesis who recovered on the 18th and 25th days after the operation by gastrointestinal decompression and nasoenteral nutrition tube placement through s gastroscope for enteral nutrition. All the above patients recovered after conservative nonoperative treatment. All 41 patients recovered well and were followed after surgery, and no serious complications or perioperative death occurred.

The patients returned for a visit on the 3rd month after surgery, and the gastroscopic results showed unobstructed anastomosis and smooth mucosa (Fig. 6). In addition, the total hospitalization cost for this group of patients was 10,935.00 ± 2205.72USD. Wang Y and others have reported that the total hospitalization cost was 14,784.00 ± 2156.00USD [14, 15]. The total hospitalization cost can be roughly divided into these parts: In-patient examination fees( physical examination and Laboratory test fees), consumable material (anastomotic materials, ultrasonic knif, Trocars, Vicryl, etc.), medicine fees, hospitalization, physicians, nursing fees, etc. The price of anastomotic consumables is very expensive, 100% of the cost need to be paid by patients, accounting for a large proportion of the total cost, while the other cost are in the scope of medical reimbursement, accounting for 60–90% of the reimbursement proportion. Therefore, saving the cost of anastomotic consumables can certainly reduce most of the cost and greatly reduce the economic pressure of patients. The results above indicate that totally laparoscopic hand-sewn Billroth I anastomosis is a safe and feasible method for digestive tract reconstruction.

### Advantages

Totally laparoscopic hand-sewn Billroth I anastomosis has the following advantages. (1) It conforms to human anatomy and physiology. Totally laparoscopic hand-sewn Billroth I anastomosis manages to save the continuity of the digestive tract and the feedback mechanism of autocrine and paracrine systems, which are more consistent with normal physiological structure. Therefore, reflux gastritis caused by bile reflux can be avoided [3]. (2) A clear field of vision during totally laparoscopic hand-sewn Billroth I anastomosis is guaranteed. Compared with other anastomotic methods, this method is limited to the right upper abdomen. There is no need to frequently change the field of vision, which can decrease the difficulty of assistant coordination. (3) The number of anastomoses is decreased, which may reduce complications such as anastomotic bleeding and leakage. Under the skilled cooperation of teamwork, the total time of the operation and time of anastomosis by hand were acceptable [13]. In addition, the operation time was gradually reduced with the extension of the learning curve (Fig. 7). CUSUM analysis shows 21 cases were the minimum number of surgeries needed to skillfully master double layered end-to-end anastomosis with continuous manual suture to complete digestive tract reconstruction across the learning curve. (4) The hand-sewn anastomosis is relatively smooth compared to anastomosis with a stapler, which is prone to having overlapped corners or “dog ears”, causing a higher risk of anastomotic leakage as well as adhesion. (5) The resection range and reduction in anastomotic tension are fully ensured. Hand-sewn Billroth I anastomosis is a standard end-to-end anastomosis, which can reserve the overlapped gastroduodenal wall wasted in triangle-shaped anastomosis with a stapler. (6) Mesenteric hiatal hernia and Petersen’s hernia can be avoided in hand-sewn Billroth I anastomosis. (7) There are decreased complications such as adhesive ileus. This procedure is limited to the right upper abdomen, which can reduce potential mechanical stimulation and mucosal damage to the jejunum therefore decreasing the incidence rate of postoperative intestinal adhesion.

### Difficulties and countermeasures

Although totally laparoscopic hand-sewn Billroth I anastomosis has many advantages, it is still a very difficult technique for some surgery teams that newly start practising totally laparoscopic hand-sewn Billroth I anastomosis. For example, it has been previously reported that intra-abdominal anastomosis can increase the incidence rate of abdominal infection [16]. In our study, a bulldog clamp was used to block the gastric and duodenal stumps to avoid intestinal fluid leakage, and this clamp was not removed until full-thickness anastomosis was completed. Additionally, the surgical team needs to discuss in advance the specific anastomosis steps in great detail to achieve perfect cooperation [17].

## Conclusions

Double layered end-to-end anastomosis with continuous manual suture is a safe and feasible method for digestive tract reconstruction in totally laparoscopic distal gastrectomy, as it not only ensures the completion of Billroth I anastomosis but also may reduce the operation cost to a certain extent. It is worth further discussion and practice, and prospective controlled studies with large samples sizes are needed.

## Data Availability

The data analyzed during the current data-analysis is available from the corresponding author on reasonable request.

## Abbreviations

TLDG: Totally Laparoscopic Distal Gastrectomy
CT: Computed Tomography
